# Mitochondrial regulation in human pluripotent stem cells during reprogramming and β cell differentiation

**DOI:** 10.3389/fendo.2023.1236472

**Published:** 2023-10-20

**Authors:** Ila Tewari Jasra, Nerea Cuesta-Gomez, Kevin Verhoeff, Braulio A. Marfil-Garza, Nidheesh Dadheech, A. M. James Shapiro

**Affiliations:** ^1^ Clinical Islet Transplant Program, Department of Surgery, Alberta Diabetes Institute, University of Alberta, Edmonton, AB, Canada; ^2^ Tecnologico de Monterrey, The Institute for Obesity Research, Monterrey, Nuevo Leon, Mexico

**Keywords:** Diabetes Mellitus, stem cells, induced pluripotent stem (iPS) cells, islet transplantation, beta cells (β Cells)

## Abstract

Mitochondria are the powerhouse of the cell and dynamically control fundamental biological processes including cell reprogramming, pluripotency, and lineage specification. Although remarkable progress in induced pluripotent stem cell (iPSC)-derived cell therapies has been made, very little is known about the role of mitochondria and the mechanisms involved in somatic cell reprogramming into iPSC and directed reprogramming of iPSCs in terminally differentiated cells. Reprogramming requires changes in cellular characteristics, genomic and epigenetic regulation, as well as major mitochondrial metabolic changes to sustain iPSC self-renewal, pluripotency, and proliferation. Differentiation of autologous iPSC into terminally differentiated β-like cells requires further metabolic adaptation. Many studies have characterized these alterations in signaling pathways required for the generation and differentiation of iPSC; however, very little is known regarding the metabolic shifts that govern pluripotency transition to tissue-specific lineage differentiation. Understanding such metabolic transitions and how to modulate them is essential for the optimization of differentiation processes to ensure safe iPSC-derived cell therapies. In this review, we summarize the current understanding of mitochondrial metabolism during somatic cell reprogramming to iPSCs and the metabolic shift that occurs during directed differentiation into pancreatic β-like cells.

## 1 Introduction

The introduction of the reprogramming factors Oct4, Sox2, Klf4, and c-Myc enable the reprogramming of adult somatic cells into induced pluripotent stem cells (iPSC) capable of self-renewal (1, 2). Autologous iPSCs hold promise for applications in precision health and regenerative medicine, disease modelling, and drug screening (3). Although remarkable progress has been made on molecular regulation and methods to achieve pluripotency and subsequent differentiation into lineage-specific cell types, little is known regarding the metabolic shifts that somatic cells must undergo to achieve pluripotency and differentiation into cell types of interest, such as β cells. Currently, the risk of teratogenicity and low reprogramming efficiency interfere with the translation of iPSC-based cell therapies into the clinic.

The process of reprogramming adult somatic cells involves transition in cellular characteristics, genomic and epigenetic remodelling in parallel with major metabolic changes to sustain self-renewal and plasticity of iPSCs (4, 5). Overall, the metabolic shift that occurs during reprogramming consists of decreasing oxidative phosphorylation (OXPHOS) while increasing glycolysis (6–8). Glycolysis is a very inefficient energy generation process; but, if the rate of metabolic flux is high enough, glycolysis can provide enough adenosine triphosphate (ATP) for fast cell multiplication. Glycolysis is also required for nucleic acid, amino acid, and lipid production. Despite the presence of oxygen and functioning mitochondria, increased aerobic glycolysis in iPSCs replicates the Warburg effect, in which cancer and pluripotent stem cells enhance their glucose absorption and lactate fermentation (9).

To date, several studies have investigated the mechanism behind the metabolic shift to glycolysis occurring during somatic cell reprogramming. Furthermore, differentiation of iPSC into specific tissues requires further metabolic adaptation. For this reason, this review article aims to summarize the metabolic transitions that occur in the process of somatic cell reprogramming to iPSC and during optimal iPSC generation and differentiation into β-like cells.

## 2 Mitochondrial characteristics of iPSC

Mitochondria play a crucial role in cellular energy production through oxidative phosphorylation (OXPHOS), generating ATP in all nucleated cells (4). They consist of the electron transport respiratory chain (ETC) complexes I-IV and the ATP synthase complex V (10). During the reprogramming of human induced pluripotent stem cells (iPSCs), changes occur in various mitochondrial characteristics, including number, mitochondrial DNA (mtDNA), shape, ultrastructure, and distribution, as part of the metabolic transition from OXPHOS to glycolysis. The subcellular composition and distribution of mitochondria are highly dynamic, and the organelles undergo turnover facilitated by selective autophagy, known as mitophagy, which eliminates faulty mitochondria through lysosomal machinery.

Mitophagy is a complex mechanism that selectively segregates damaged or depolarized mitochondria into double-membraned autophagosomes. These autophagosomes are then degraded by lysosomal enzymes. Mitophagy is essential cellular process that plays a significant part in cellular reprogramming because it removes undesired and damaged mitochondria or helps reorganizing mitochondrial network needed for optimal differentiation (11). During differentiation, mitochondria in iPSCs undergo mitophagy cleanup and are then replenished by newly developed mitochondria for adapting to the metabolic needs of the differentiating cells. For example, both somatic cell reprogramming into iPSC and β cell differentiation need NIX-mediated mitophagy (12). Other proteins, such as PINK1 and Parkin (Park2), are known to play important roles in controlling mitophagy in beta cells (13). PDX1, a pancreatic differentiation master regulator transcription factor, has recently been shown to regulate mitophagy in β cells (14). The balance between organelle fusion and fission processes helps maintain the adaptability of the mitochondrial network (15, 16). Mitochondrial fusion has been demonstrated to be a viable method for stress reduction in biological systems. Mitochondria that have been partially damaged can benefit from fusion because it allows for the complementation of their contents. Fission, on the other hand, is critical for the genesis of new mitochondria, but it also serves to maintain quality control by allowing the removal of defective mitochondria. Fission can potentially promote apoptosis in conditions of high cellular stress.

**Figure 1:**
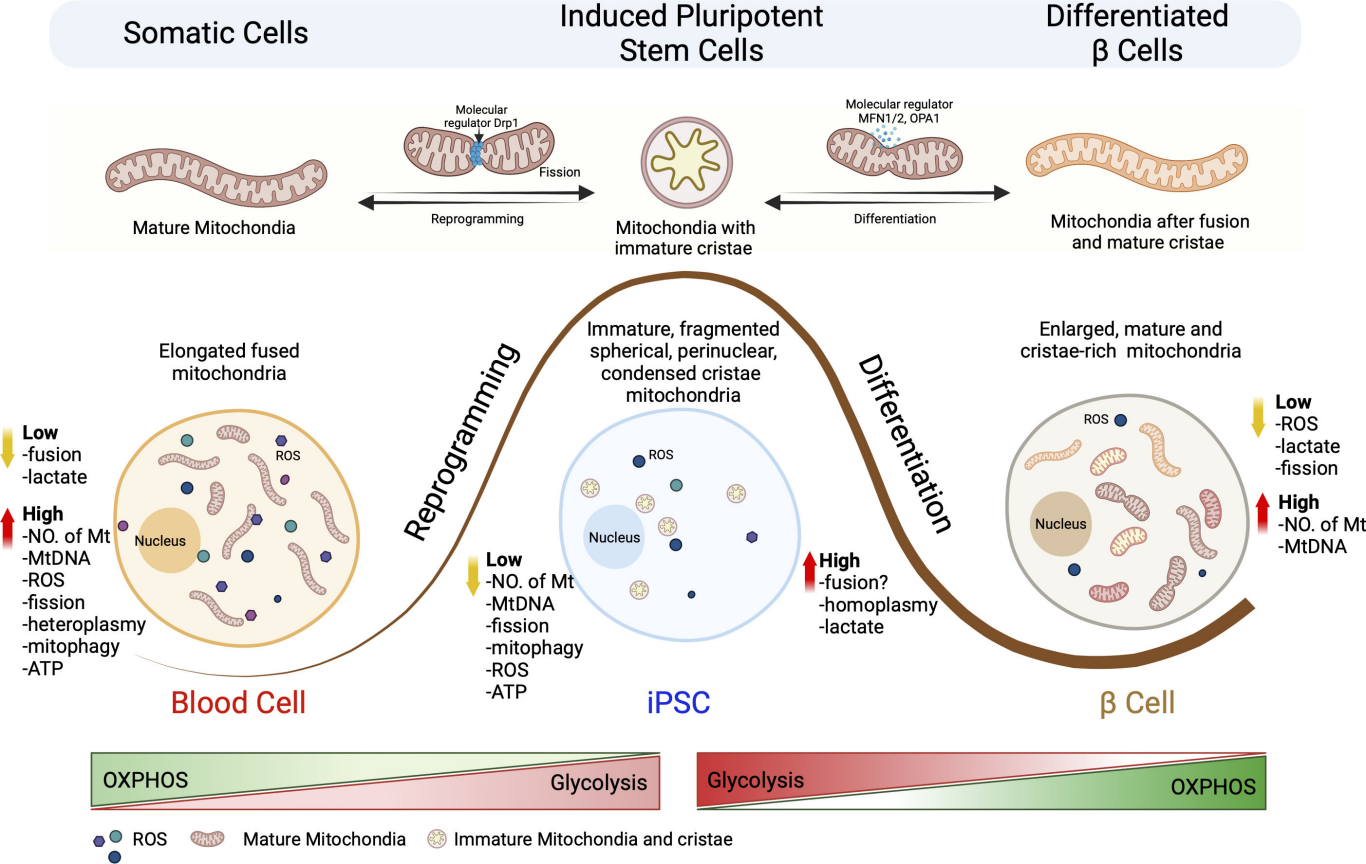
Schematic representation of the mitochondrial architectural changes during somatic cell reprogramming and differentiation of iPSCs into β cells. Somatic cells undergo reprogramming to generate iPSCs, which can be re-differentiated into specialized terminal cells. This process leads to changes in the metabolic signature for mitochondria size, number, shape, fragmentation pattern (fission vs fusion), mtDNA homo/heteroplasmy, oxidative stress and metabolic pathways. Somatic cells are characterized by the presence of high number of elongated mitochondria which have a highly active OXPHOS metabolism that generates high concentrations of ROS. Reprogramming results in fission of the mitochondria that results in immature, fragmented, spherical, and perinuclear mitochondria with condensed cristae. Differentiation of iPSCs to terminal β cells requires the fusion of mitochondria to generate large numbers of enlarged mature and cristae-rick mitochondria resulting in a transition from glycolysis to OXPHOS.

Undifferentiated embryonic stem cells (ESCs) have been found to possess a small number of mitochondria with poorly formed cristae and a perinuclear location (17). ESCs also exhibit a low number of mtDNA copies, but this increases with differentiation and mitochondrial maturation (18). In the case of iPSCs, studies using the mitochondrial dye MitoTracker Green have shown that active mitochondria are primarily found in cells along the boundaries of iPSC colonies, which reflect cells undergoing spontaneous differentiation (10, 18). This supports previous findings that undifferentiated iPSCs have fewer active mitochondria (6, 19). In contrast to the elongated, tubular-shaped, branched, and cristae-rich mitochondria found in somatic cells, iPSC mitochondria are round, globular, spherical, with underdeveloped cristae and a perinuclear distribution (**Figure 1**). The number and mass of mitochondria are also lower in iPSCs compared to their original somatic cells (18, 20). These observations have led some scientists to propose that the reduction in mitochondria may be associated with mitophagy-like processes induced during the reprogramming process (13, 21, 22). In fact, the autophagy stimulator- rapamycin has been shown to enhance iPSC reprogramming efficiency (20, 23), suggesting a potential link between mitophagy activation and the reduction in mitochondria during reprogramming (24). Rapamycin is a mTOR complex inhibitor that binds to mTOR1 complex and promotes mitophagy and cell survival in response to ER stress (25, 26). It plays an important role in identifying and degrading damaged mitochondria by inducing the expression of genes promoting mitophagy (*PINK1, PARKIN, ULK1, AMBRA1*) and mitochondrial fission (*FIS1, DRP1*) *(* 27).

It is important to note that the mitochondrial characteristics observed in iPSCs are transient and change as the cells differentiate (28, 29). With differentiation into fibroblast-like cells, iPSCs exhibit an increase in mtDNA copy number and undergo mitochondrial maturation, acquiring tubular structures with well-formed cristae, similar to somatic fibroblasts (30). These findings highlight the adaptability of iPSCs and their mitochondria and have sparked considerable interest in the field in characterizing iPSCs for their function and mitochondrial metabolism.

In conclusion, the characteristics of mitochondria in iPSCs undergo changes during reprogramming, including alterations in number, mtDNA, shape, ultrastructure, and distribution but remain stable during prolong cell generations, as connected with the pluripotency state. Mitochondrial characteristics associated with the metabolic transition from OXPHOS to glycolysis undergo substantial changes only upon directed differentiation only. Thus, understanding the functional and metabolic implications of these mitochondrial features in iPSCs, while maintaining pluripotency and upon differentiation, is an important area of research that can provide clear insights into efficient cellular reprogramming and differentiation processes.

## 3 Metabolic features of iPSC

When compared to somatic cells, iPSCs have a higher rate of proliferation and diverse cell cycle properties, such as a shorter G1 phase and a longer S phase (31). As a result, a significant portion of cellular energy is committed in constructing the biomass required for ongoing cell development while maintaining the undifferentiated state. In addition, pluripotency maintenance by exogenous pluripotency-associated transcription factors, need greater anabolic requirements to carry out significant chromatin state, gene expression, and signal transduction modulation, allowing somatic cells conversion into iPSCs (32).

**Figure 2:**
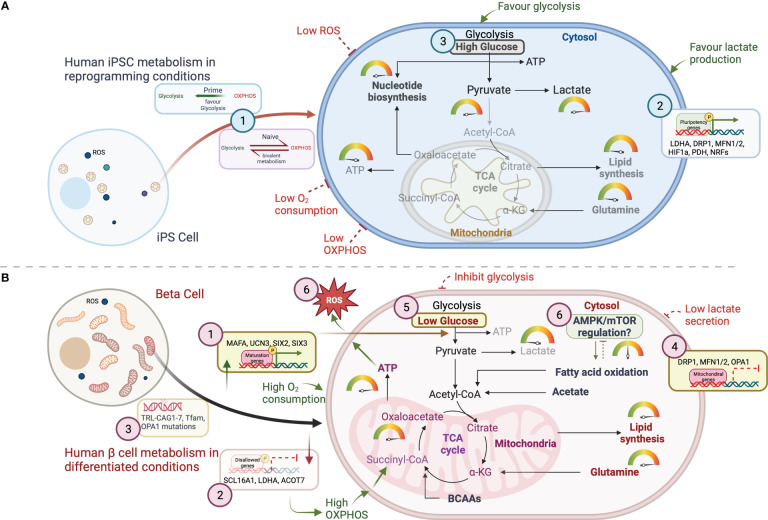
Schematic representation of the key metabolic mechanistic pathways for energy generation in **(A)** iPSCs and **(B)** β cells. Glycolysis breaks down glucose into two molecules of pyruvate which can enter the mitochondria upon oxidative decarboxylation into acetyl CoA in the mitochondrial matrix. Acetyl-CoA then enters in the TCA cycle where it is oxidized for energy production and/ or for the generation of metabolic intermediates for fatty acid and nucleotide biosynthesis. Metabolic intermediates that arise throughout glycolysis, including glucose-6-phosphate, fructose-6-phosphate, and dihydroxyacetone phosphate (DHAP), provide the scaffolds for fatty acids and amino acids synthesis required to support the increasing biomass. Several key molecular mechanisms regulating the mitochondrial metabolic homeostasis are represented with numbers in this figure. **(A)** For iPSCs, three mechanisms are summzied as (1), pluripotency phenotypes (naïve vs prime)- responsible for metabolic shift between glycolysis and OXPHOS (2); mitochondrial genes affecting pluripotency genes expression to help regulating optimal glycolytic function in iPSCs; (3) Nutritional requirements facilitating glycolytic pathway for lactate production. **(B)** Mechanisms in human β cells include, (1) islet maturation associated genes contribute to achieve metabolic homeostasis; (2) repression of disallowed genes improves mitochondrial function and high ATP production; (3) screening mtDNA mutations will further help eliminating the risk of mitochondrial dysfunction; (4) genomic regulation of mitochondrial genes; (5) Nutritional control for ATP production and maturation; and (6) increased ROS production as a result of high mitochondrial function and respiration.

Glycolysis provides more than just ATP for fast growth. Furthermore, glycolytic pathway intermediaries supply precursors for fatty acid and amino acid synthesis, which are essential to sustain iPSC biomass (15). Even in the presence of oxygen, iPSCs preferentially convert glucose to lactate, just like growing tumor cells (33). This glycolytic state is linked to the cellular reprogramming process, which ensures the appropriate ATP demand required for pluripotency. Indeed, bioenergetic investigations have revealed that, as compared to somatic cells, iPSCs create a significant quantity of lactate while producing less ATP and consuming less oxygen, which fits with the few immature mitochondria observed on iPSCs (**Figure 2**) (34). iPSCs, on the other hand, primarily oxidize glucose *via* cytosolic glycolysis to create ATP, whereas somatic cells have highly structured, cristae-rich, and respiratory-active mitochondria to feed their energy demands *via* OXPHOS. As a result, mitochondrial ultrastructure, number, and resting mitochondrial membrane potential (ΔΨ) in iPSCs are significantly different. Despite mitochondrial immaturity, ΔΨ plays an important role in mitochondrial homeostasis by selectively eliminating defective mitochondria (35). Low mitochondria number with reduced ΔΨ and oxidative capacity, can explain for glycolytic metabolism of stem cells. Changes in ΔΨ is suggested to be a predictive indicator of self-renewal, lineage commitment and to select optimal stem cell population with enhanced pluripotency and differentiation potential. Recently, Woods and colleagues performed a metabolic study with pluripotent stem cells to show that stem cells with lower average ΔΨ more efficiently drive into primordial germ-like cells (36). In culture, human iPSCs are discovered to inherit higher ΔΨ, and this hyperpolarization is thought to be a result of decreased ATP generation *via* OXPHOS but more pronounced glycolysis-based energy flux (33, 37). High ΔΨ in cells allow them to handle the rising energy demands for stemness and pluripotency, where the mitochondrial ATP synthase hydrolyzes the ATP generated from glycolysis and supply the instant energy demand for early differentiation or lineage specification. Despite immaturity, mitochondria in iPSCs are still capable of producing sufficient ATP from OXPHOS and consuming oxygen (34, 38).

Specific transcriptional fingerprints can also be used to identify an iPSC's metabolic profile. Genes regulating gluconeogenesis, glycolysis's first and last steps, and the non-oxidative branch of the pentose phosphate pathway (PPP) are upregulated in iPSCs compared to somatic fibroblasts, favoring glycolysis and the diversion of glycolytic intermediates into the PPP, which provides nucleotide precursors necessary for anabolic growth (38). Metabolite analysis indicated that iPSCs accumulate more glucose-6-phosphate than somatic fibroblasts, indicating PPP activation (34, 38).

The glycolytic state of iPSCs may be phenocopied mechanistically by inhibiting pyruvate transport into mitochondria (pyruvate shunting), resulting in decreased availability of pyruvate as a substrate for the tricarboxylic acid cycle (TCA) and increased conversion of pyruvate to lactate in the cytosol (37, 39). As a result, 3-Phosphoinositide-dependent kinase1 (PDK1), a small molecule activator has been found to greatly improve the effectiveness of cellular reprogramming (39) . Furthermore, iPSCs may have significant levels of oxidized pyruvate kinase isoform M2 (PKM2), which has reduced catalytic activity. Because of the decreased catalytic activity, reduced phosphoenolpyruvate is converted to pyruvate, shifting energy flow beyond the mitochondria to upstream glycolysis-related processes (40).. In both normoxic (21% O2) and hypoxic (1-5% O2) circumstances, iPSCs prefer glycolysis over OXPHOS. Although, iPSCs thrive better in hypoxic condition, despite having a 19-fold better ATP production efficiency than glycolysis, iPSCs are unable to adopt OXPHOS-based metabolism in the absence of accessible oxygen (5). It has been proposed that higher ATP levels in iPSCs, irrespective of maintenance oxygen circumstances, allow cells to undergo glycolysis. It has also been shown that lack of oxygen during reprogramming may impede mitochondrial remodelling, preventing cells from achieving higher levels of OXPHOS in iPSCs (41).

Mitochondria are the principal source of harmful reactive oxygen species (ROS), which are common by-products of OXPHOS (42). ROS can then cause oxidative damage to DNA, proteins, or lipids, which antioxidant enzymes can prevent (43). If this regulated redox equilibrium (ratios of reduced/oxidized cofactors and proteins- NAD(P)H/NAD(P)+ and GSH/GSSG) is disrupted, oxidative stress can develop, resulting in negative cellular consequences, a circumstance that is expected to occur during the ageing process and age-related neurodegeneration (44). As a result, in the context of redox balance, iPSC glycolytic metabolism offers additional benefits. To begin with, it reduces ROS generation by decreasing mitochondrial respiration. Second, by increasing the flow *via* the PPP's oxidative branch, it supplies the reducing component nicotinamide adenine dinucleotide phosphate hydrogen NADPH, which is essential for antioxidant enzyme activity (45).

To ensure extended cell survival and proliferation capacity, iPSCs increase their stress-protective mechanisms and acquire a strictly restrained redox state *via* upregulation of antioxidant enzymes such as uncoupling protein 2 and superoxide dismutase 2, resulting in low levels of oxidatively-modified proteins, lipids, and DNA, ensuring maximum safeguard against genome instability in the undifferentiated state (20). As a result, it has been proposed that the cellular metabolome of iPSCs has an abundance of unsaturated metabolites that are extremely vulnerable to oxygenation and hydrogenation processes (18), enabling iPSCs to adapt swiftly to increased metabolic oxidation by initiating differentiation programmes, a mechanism known as "chemical plasticity". In this process, undifferentiated stem cells attain their low oxidative state of metabolomes, which are concentrated in highly unsaturated metabolites. Once differentiated, such metabolites decreased dramatically with a marked increase in newly hydrogenated and oxygenated compounds. The phenomenon is an akin to cell plasticity, and therefore known as chemical plasticity (46). It is worth noting that the addition of certain cell stage-specific metabolites or modifications of related metabolic pathways consistently promoted stem cell self-renewal or facilitate specific differentiation, highlighting the role of metabolic products in defining cell fate.

Human iPSCs show high levels of mitochondrial uncoupling protein UCP2 compared to somatic cells, which regulates glutamine and glucose oxidation for self-renewal capacity (47). High-lipid supplements are essential for iPSC self-renewal and glycolytic mode of action and should be removed during cell differentiation. DRP-1 protein inhibition in mitochondria facilitates early reprogramming process by increasing reduced expression-1 (REX1) expression to maintain pluripotency. In somatic cell reprogramming, MAPK inhibition is required for DRP1 phosphorylation *via* ERK pathway. Particularly, depletion in fusion protein Mfn1/2 plays key mechanistic role in OXPHOS transition into glycolytic states followed by ROS mediated HIF1α activation to favor hypoxic reprogramming. During differentiation, DRP1 activity plays a crucial mechanism in cellular lineage patterning for cardiac, muscle, and neuronal cells (48–50). However, in islet cell differentiation, Mfn1/2 and OPA1 play central roles, instead of DRP1, to switch from glycolysis to OXPHOS. Additionally, ROS generation during cell differentiation alters DRP1 and Fis1 expression leading to increased mitochondrial fission. The mechanism associated in the process requires recruitment of protein disulfide isomerase A1 (PDIA1), OPA1, and Sirtuin 4 to induce fission while regulating mitophagy (51).

To summarize, iPSCs utilize glycolysis as their main pathway for energy production, and primarily dependent on fatty acid or amino acid precursors for their metabolic demands to achieve unrestrained expansion. Furthermore, the preference of glycolysis over OXPHOS results in a reduction of ROS generation, which in combination with increased stress defense mechanisms, results in extended cell survival with maximal safeguard from genome instability (52).

## 4 Mitochondrial physiology during reprogramming and differentiation

Cellular reprogramming requires extensive mitochondrion architectural reorganization and metabolic shift to balance the need for pluripotency and self-renewal. In this section we describe key morphological, genetic, or epigenetic factors and metabolic states that regulate iPSC reprogramming and terminal cell differentiation.

### 4.1 Morphological and numerical changes of mitochondria and subunit composition

The role of mitophagy and mitochondrial dynamic fission is critical process of reprogramming somatic cells into iPSCs. During reprogramming, mitochondria adopt a rejuvenated state (condition of attaining youthful characteristics after somatic reprogramming) (33, 53) through elusive mechanisms that include mitochondrial fission and mitophagy (21). It suggests that the autophagy mediated regulation of mitochondrial dynamics can selectively target dysfunctional mitochondrial clearance, leading to cellular changes such as increased glucose uptake and a shift from mitochondrial respiration to glycolysis. This metabolic shift is important for achieving pluripotency. Mitophagy facilitates glycolysis which is not as result of mitochondrial biogenesis but attributed to increased formation of autophagic acidic vesicles enclosing the dysfunctional mitochondria. Studies also suggest that autophagy inducers and mTOR inhibitors can enhance the efficiency of iPSC generation without compromising pluripotency due to mTOR-regulated changes in mitochondrial fission and fusion. The process of mTOR complex inhibition controls size, morphology, and number of mitochondria suggesting that increased mitophagy enable successful somatic reprogramming while mitophagy abrogation leads to cell senescence.

The mechanism for the selective clearance of defective mitochondria was first described by Fan et al. in their initial study (54). Later, Pei et al. screened all four Yamanaka factors (Oct4, Sox2, Klf4, and c-Myc) and discovered that they all work together to suppress mTOC1. However, the study outlined specific its mechanism mediated *via* Klf4 and c-Myc factors restraining mTOR complex to activate ATG5-dependent autophagy while Sox2 and Oct4 repress mTOR1 and bifurcate in autophagy-related gene suppression (*Ulk1* and *Atg* genes) (55). Furthermore, Ding et al. showed that an ATG5-independent autophagic pathway is indispensable for iPSC induction, since inhibition of ATG-5 dependent canonical pathway inhibits autophagy of mature mitochondria which is critical for the metabolic shift (56). Although the relationship between the pluripotency reprogramming factors and the autophagy pathway genes remain controversial, further investigations are required to descend the observed differences in somatic reprogramming and directed differentiation methodologies. Overall, the published studies supported the notion that a decline in mitochondrial number may be vital for iPSC reprogramming.

Furthermore, there is also a reduction in mitochondrial size through mitochondrial fission. c-Myc, an important reprogramming factor, elicits the activation of DRP1, which induces active fragmentation resulting in smaller mitochondria with high OXPHOS. Inhibition of DRP1 (21) or induction of mitochondrial fusion proteins MFN1 and MFN2 suppresses reprogramming (57), while inhibition of MFN1 and MFN2 increases reprogramming efficiency by enabling HIF1α stabilization and facilitating the transition from OXPHOS to glycolysis (57). Similarly, Klf4, also lowers the mitochondrial content and OXPHOS during reprogramming through inhibition of mitochondrial polynucleotide phosphorylase (PnPase) (58, 59) . PnPase maintains mitochondrial homeostasis through facilitating the import of nucleus-encoded RNA required for mtDNA replication, transcription and translation (60) . Klf4 regulates PnPase through T-cell leukemia/ lymphoma 1 (*Tcl1*), which is induced during late reprogramming and suppresses the activity of PnPase lowering the mitochondrial content and OXPHOS (5).

To facilitate the energetic shift, during early reprogramming stages the expression of the subunit proteins in complexes I and IV is downregulated while there is an increased expression of proteins in complexes II, III and V of the electron transport chain (61). Alterations in the stoichiometry of the different subunits results in functional changes of the mitochondria. The low expression of complex I and high expression of complex II enhances the reduction of flavin adenine dinucleotide (FAD^+^) to FADH2 over the reduction of nicotinamide adenine dinucleotide (NAD^+^) to NADH, which in combination with decreased expression of complex IV, reduces O_2_ to H_2_O. Resulted transient burst of OXPHOS activity in turn promotes glycolysis and the metabolic shift (62).

### 4.2 Upregulation of glycolysis

Upregulation of proteins involved in glycolysis occurs early and is maintained throughout the reprogramming process up until the generation of established iPSCs (33). This upregulation of glycolytic enzymes is consistent with an increase in lactate production (58, 63). Hypoxia-related genes Hif1ɑ and Hif2ɑ are upregulated during the early stages of reprogramming because of increased glycolysis. In early stages of reprogramming, Hif1ɑ and Hif2ɑ increase the expression of glycolytic enzymes, while at later stages Hif2ɑ induces apoptosis through tumor necrosis factor-related apoptosis-inducing ligand (64).

Glycolysis is not only favored by upregulation of the glycolytic enzymes, but also by post-transcriptional regulation of other key metabolic enzymes. Pyruvate dehydrogenase (PDH) connects glucose and fatty acid oxidation by converting pyruvate to acetyl-CoA, augmenting the input of acetyl-CoA from glycolysis into the TCA cycle. The protein levels of pyruvate dehydrogenase kinase 1 (PDHK1), a negative regulator of PDH activity through phosphorylation, are increased during reprogramming, which decreases conversion of pyruvate into acetyl-CoA and hence, favoring the generation of lactate (5). Reprogramming induces the generation of PKM2 instead of PKM1 *via* alternative splicing (65). Pyruvate kinase catalyzes the generation of pyruvate from phosphoenolpyruvate, and PKM2 has lower catalytic activity than PKM1, decreasing the generation of pyruvate available for OXPHOS and hence, enhancing glycolytic activity (66).

At later stages of reprogramming, core pluripotency factors Oct4, Sox2, Nanog, c-Myc, are directly involved in the maintenance of aerobic glycolysis. In reprogrammed stem cells, the core pluripotency transcription factors, commonly referred to as CPTFs, interact with the promoters of genes encoding glycolytic enzymes, namely hexokinase 2 (HK2) and pyruvate kinase M2 (PKM2), to effectively drive transcription and significantly enhance glycolytic flux. The metabolic coordination exhibited by CPTFs strongly suggests their indispensable role for modulating mitochondrial metabolism controlling pluripotency (67). Oct4 directly upregulates the expression of hexokinase (HK)-2, and PKM2 (68). HK generates glucose-6-phospate in the first step of glycolysis, which combined with a low conversion of phosphoenolpyruvate to pyruvate, maintains a high glycolysis rate (68). The inhibition of HK2 produces a metabolic shift from glycolysis to OXPHOS resulting in the loss of pluripotency (69). Furthermore, through Tcl1 expression induction, Klf4 directs metabolic shift during reprogramming by (a) enhancing glycolysis and (b) diminishing OXPHOS. Tcl1 stimulates protein kinase B, also known as Akt, that promotes glucose metabolism by enhancing glycolysis and inhibits PnPase to diminish OXPHOS (58).

### 4.3 Remodelling of alternative mitochondrial metabolic pathways

Despite the switch from OXPHOS to a highly glycolytic state being a driving force for reprogramming (33), adaptations in lipid and amino acid metabolism pathways are also relevant during nuclear reprogramming (8). *De novo* fatty acid biogenesis promotes somatic cell reprogramming efficiency and pluripotency maintenance by regulating mitochondrial fission. Nuclear reprogramming enhances the expression of acetyl-CoA carboxylase 1 (ACC1), which catalyzes the irreversible carboxylation of acetyl-CoA to malonyl-CoA, the building block of fatty acid synthesis (70). In this manner, ACC1 decreases the availability of acetyl-CoA in the cytoplasm, which promotes the degradation of mitochondrial fission 1 protein (Fis1), and thus, decreases mitochondrial fission. However, increased *de novo* fatty acid synthesis decreases cellular acetyl-CoA levels, resulting in stabilization of Fis1 and increased mitochondrial fission which enhances reprogramming (21). In addition, nuclear reprogramming induces the upregulation of caritinine palmiltoytransferase 1B, which transfers the acyl group from coenzyme A to carnitine, enabling the transfer of long-chain fatty acids into mitochondria. Addition of palmitoylcarnitine, the product of caritinine palmiltoytransferase 1B, stimulates OXPHOS during early reprogramming, which fosters a transient hyper-energetic state (71). At later stages of reprogramming, palmitoylcarnitine does not increase OXPHOS but generates acetyl-CoA inside the mitochondria, which is converted into citrate by-citrate synthase and exits the mitochondria generating cytosolic acetyl-CoA, which maintains the open state of the chromatin structure, and thus, pluripotency through acetylation of histones (71). Here we described alternative mitochondrial metabolic pathways that induce reprogramming other than previously mentioned. Although contradicting to mitochondrial fusion, iPSC in certain specific culture conditions could attain stemness and somatic reprogramming when treatment differently, early-on.

Excessive glycolysis is associated with the accumulation of advanced glycation end products (AGEs), including methylglyoxal-derived AGEs, which are associated with several degenerative disorders including diabetes, chronic kidney disease and Alzheimer’s disease (17). However, despite the metabolic switch from OXPHOS to glycolysis, the number of methylglyoxal-derived AGEs accumulated in somatic cells gradually decreases during reprogramming until it is found absent in established iPSCs (72). During early stages of cellular reprogramming, Klf4 and c-Myc induce the expression of glycine decarboxylase (GLDC), the rate limiting enzyme in the serine/glycine biosynthesis pathway, where 3-phosphoglycerate is used for the generation of serine and glycine. GLDC promotes the upregulation of glycolysis and the serine/glycine pathway while suppressing the generation of methylgloxal-derived AGEs (72). Upregulation of GLDC promotes reprogramming while inhibition of GLDC induces the accumulation of AGE in iPSC and reverses pluripotency, showing the relevance of amino acid metabolism in induction and maintenance of pluripotency (73).

### 4.4 Genetic and epigenetic regulation of mitochondrial metabolism

Functional potential of iPSC-derived cells is suggested to be closely linked to mitochondrial genome, epigenetic regulation and mtDNA heteroplasmy (24, 74). iPSC reprograming remodels the existing epigenetic memory and establishes a new epigenetic profile that complies iPSC identity and pluripotency (75). The activity of mitochondrial metabolites produced by the TCA cycle regulate DNA methylation and chromatin changes during cellular reprogramming. It has been suggested that somatic reprogramming is associated with improved expression of acetyl-CoA, -ketoglutarate, NADH/NAD pool, and TCA cycle product such as citrate and succinate (54). The metabolites, in turn, control chromatin machinery involved in development and proliferation *via* acetylating histones proteins (49, 55). Acetyl-CoA supplies the requisite acetyl groups to initiate the reaction specifically required for histone acetylation process carried out by histone acetyltransferases (HATs). HATs are responsible for addition of acetyl groups in histone N-terminal trials and alter the dynamics of chromatin to drive the epigenetic control of gene expression by activating transcriptional programs. They are highly dependent on glucose availability, fatty acid oxidation and mitochondrial respiration. Mitochondrial TCA cycle acetyl-CoA generating enzyme- ACLY is responsible for cytosolic entry of citrate to synthesize acetyl-CoA which directly controls the histone modification process. Further, histone demethylases like Jumonji C domain-containing (JMJD) family histone demethylases and ten-eleven translocation (TET) DNA demethylases, as well as α-ketoglutarate (αKG), all require this component. αKG provides a substrate for chromatic modifying enzymes and its availability has a direct impact on gene expression for cellular fate decisions by regulating histone and DNA demethylases (76). In addition, succinate, a by-product of 2-oxoglutarte dependent dioxygenases (2-OGDD) enzyme processes from αKG, when it builds up, it acts as an antagonist to the reaction. Through the inhibition of histone and DNA demethylases, 2-HG and fumarate can also change the way that cells express their epigenetic information. Notably, the largest histone variation- macroH2A has a reductional explanation that controls this specific epigenetic signature to iPSCs during the somatic state to pluripotency state transition (61).

Furthermore, ROS and the master regulator of cellular resistance to oxidants and activates nuclear factor erythroid 2–related factor 2 (*Nrf2*) which play an important role in the epigenetic remodelling of iPSCs during reprogramming (5). High amounts of ROS generated from OXPHOS results in HIF1α induction which serve as a signal to modify cysteine residues on Kelch like ECA associated protein 1 (Keap1). Keap1 is an adaptor subunit of Cullin 3-based E3 ubiquitin ligase and directly regulates the activity of Nrf2 to acts as a sensor for oxidative and electrophilic stresses, KEAP1, the repressor of Nrf2, resulting in Nrf2 activation, and the subsequent interaction with HIF1α to induce the metabolic shift towards glycolysis as well as PPP and increase in nucleic acid synthesis (4). Furthermore, Nrff2 is a pivotal regulator of self-renewal, proliferation, and differentiation where High levels of ROS will modify KEAP1 and NRF2 activity to allow them freely to interact with bind Oct4 and Nanog promoters for self-renewal induction while maintaining pluripotency (77). The key players in the process are c-Myc, Lin28, PARP1, and ROS associated genes such as GSR, SOD2, MGST1 and MAPK26 (74). During differentiation, the metabolic shift resulting in increased ROS and DNA damage controls macroH2A1 expression and suppresses PARP1, which is under control of cMyc. PARP1 inhibition promotes the methylation of Sox2, Oct4 and Nanog promoters, inhibiting their transcription and hence, promoting differentiation.

### 4.5 Transient hyper-energetic state

Somatic cells that are highly reliant on glycolysis appear to undergo more efficient reprogramming than cells with an OXPHOS phenotype (40). Moreover, induction of glycolysis enhances reprogramming while induction of OXPHOS or inhibition of glycolysis hampers this process (33), suggesting that the metabolic shift is not a consequence of reprogramming but a driving force. It has been suggested that metabolic changes during reprogramming are driven by shift in metabolic demands. However, while examining gene expression patterns, scientists discovered that early in the reprogramming process, both OXPHOS and genes related to glycolysis reached their highest levels of expression (78). The presence of mitochondrial proteins and OXPHOS increases by day three of reprogramming (79) resulting in a transient hyper-energetic state where cells show both increased glycolysis and OXPHOS (80). c-Myc plays an essential role for the induction of this transient hyper-energetic state by strongly upregulating the estrogen-related nuclear receptors (ERR) ERRα and ERRβ, which control the expression of genes involved in the TCA and OXPHOS (81). Estrogen-related nuclear receptors (ERR) are a group of nuclear receptors involved in regulating genes related to energy metabolism and mitochondrial function. ERRα (Estrogen-related receptor alpha) and ERRβ (Estrogen-related receptor beta) are two members of this receptor family. While their name suggests a connection to estrogen, they do not bind estrogen like classical estrogen receptors. Instead, they are considered orphan receptors because their exact endogenous ligands (activating molecules) are not well-defined.

ERRα and ERRβ primarily regulate genes involved in energy metabolism, mitochondrial biogenesis, and oxidative phosphorylation. They help coordinate the expression of genes related to processes like glucose and fatty acid metabolism, making them important for cellular energy production. These receptors can respond to changes in metabolic demands within cells and are also involved in the development of various tissues and organs. Their activity is influenced by co-regulators and environmental factors, allowing them to adapt to different cellular contexts.

c-Myc alone can produce as robust OXPHOS as the combination of Oct4, Sox2, Klf4 and c-Myc together (81). Cells that achieve a transient hyper-energetic state possess high ΔΨ, which is a strong indicator of cells that will successfully acquire pluripotency (81). Despite the role of a transient hyper-energetic state in the metabolism switch, this state causes DNA damage, especially *de novo* copy number variations, and is detrimental for the genomic stability of the cells (82). However, we hypothesize that increased OXPHOS at the beginning of reprogramming is essential to promote the activation of Nrf2 and induce HIF1 and the subsequent transcription of glycolytic and PPP genes required to achieve and sustain pluripotency.

## 5 Role of mitochondria in glucose-stimulated insulin secretion in healthy and diseased β cells 

Pancreatic β cells adjust insulin secretion in response to circulating blood glucose, which is controlled by the equilibrium between glycolysis and OXPHOS. Disruption of OXPHOS results in impaired glucose stimulated insulin secretion (GSIS), showcasing the essential role of mitochondrial metabolism in the orchestration of β cell function (73).

In healthy, non-diabetic β cells, glucose sensing and insulin secretion is controlled by the glucokinase and the ATP production through OXPHOS (83). Briefly, glucose transporter (GLUT) 1 facilitates the diffusion of glucose into the cytosol of β cells, which gets phosphorylated by glucokinase, initiating glycolysis and generating pyruvate (84, 85). Two molecules of pyruvate per molecule of glucose are then imported into the mitochondria and into the TCA, initiating the transfer of electrons generated throughout the TCA to the respiratory chain *via* NADH and FADH_2_ and the subsequent OXPHOS mediated ATP production. Mitochondrial phosphoenolpyruvate carboxykinase (PEPCK-M) converts malate into phosphoenolpyruvate, which re-enters into TCA to sustain the mitochondrial metabolism and OXPHOS for continued ATP production (86). PEPCK-M activity depends on mitochondrial GTP generated because of the activity of the GTP-specific isoform of succinyl CoA-synthetase (SCS-GTP) (87). In mammals, SCS is a heterodimer and is located within the mitochondria. Succinyl-CoA synthetase (SCS; also known as succinate-CoA ligase) is responsible for breaking down succinyl-CoA to succinate and CoA, accompanied by the phosphorylation of NDP (ADP or GDP) to NTP (ATP or GTP) in the citric acid cycle. ATP production results in an increased cytosolic ATP/ADP ratio, promoting the closure of ATP dependent K^+^ channels and hence, the depolarization of the mitochondrial membrane that induces the influx of Ca^2+^ into the cytoplasm through voltage dependent Ca^2+^ channels. It is the influx of cytosolic Ca^2+^ upon initiation of glycolysis by the glucokinase what triggers insulin secretion (88, 89). In the presence of low concentration of blood glucose, K^+^ channels in the cell membrane are open and Ca^2+^ channels are closed, preventing insulin release. Under these circumstances, fatty acid oxidation generates enough ATP to maintain basal levels of insulin secretion (88).

In diabetes, metabolic disfunction impairs glucose sensing, compromises mitochondrial regulation, lowers ATP production and ultimately, impacts insulin secretion (90). Approximately 1% of all types of diabetes are caused by mtDNA mutations (91) and to date, there are 54 known mutations associated to diabetes pathology and prognosis (92). Mutations of TFAM, which regulates the transcription and replication of mitochondrial DNA, result in decreased hyperpolarization of the mitochondrial membrane, resulting in reduced Ca^2+^ influx and hence, suboptimal GSIS (91, 93). Mutations associated to decreased oxygen consumption, mitochondrial ATP generation, diminished glucokinase activity or mitochondrial ΔΨ all result in dysfunctional GSIS (94). Furthermore, morphological impairment of β cell mitochondria is also associated with diabetes. Fusion or fragmentation of the filamentous network of mitochondria found on healthy β cells impairs GSIS (95, 96).

## 6 Metabolic alterations during iPSC-derived β cell differentiation

**Figure 3:**
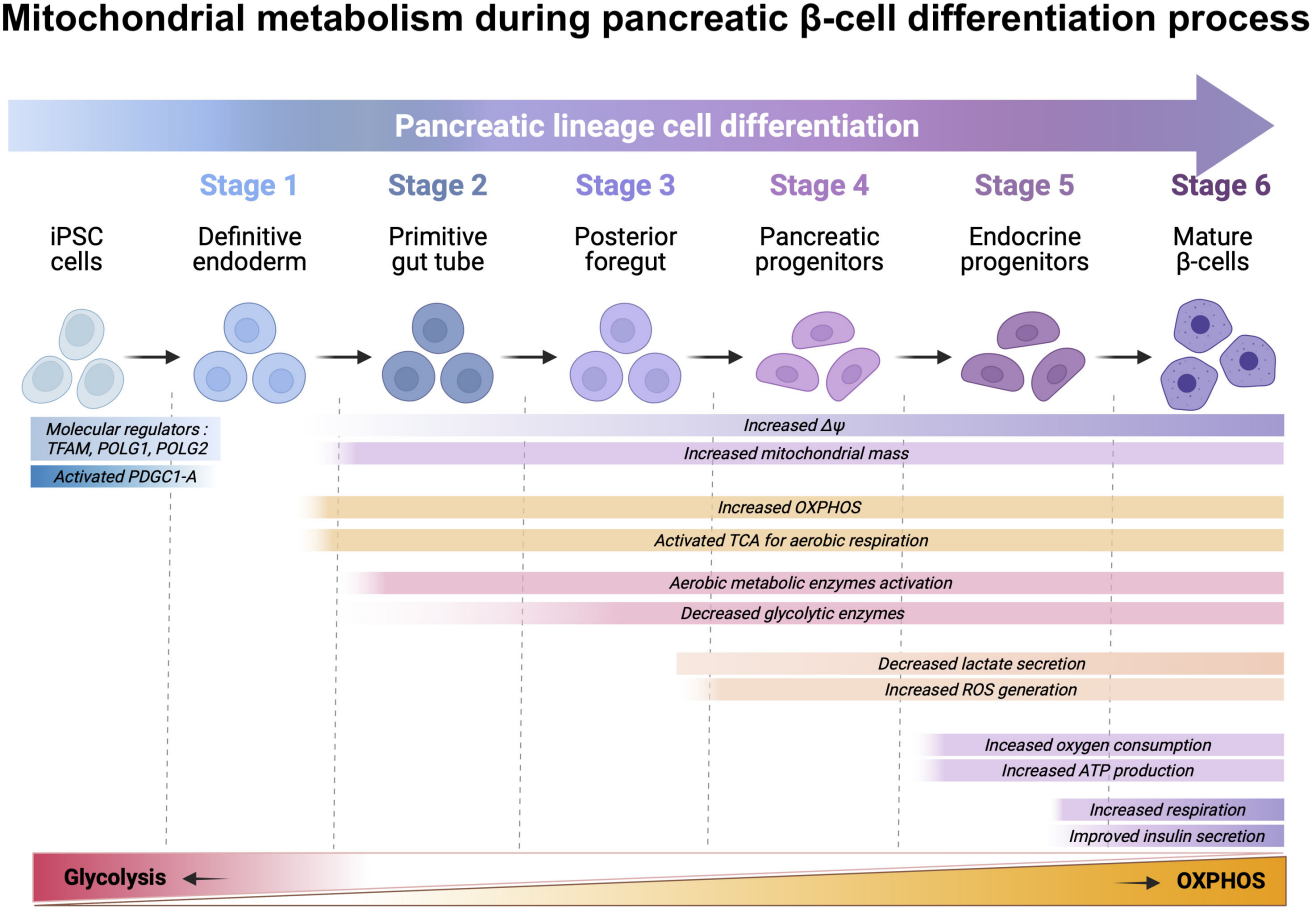
Schematic representation of directed differentiation of iPSC towards β cells and the metabolic shift that happens throughout the process. iPSCs can be differentiated *in vitro* β cells by inducing iPSCs into definitive endoderm (DE) (stage-1), which are further differentiated into primitive gut tube (PGT) (stage-2) and posterior foregut (PFT) (stage-3) before developing as pancreatic progenitors (PP) (stage-4). PPs can be further differentiated *in vitro*, into pancreatic endocrine precursors (PEPs) (stage-5) and lastly, into β cells (stage-6). Differentiation of iPSC towards β cells include a variety of changes for increased mitochondrial mass, mitochondrial membrane potential, ATP production, intracellular ROS level, and regulation of anaerobic and aerobic metabolic-associated genes. Briefly, differentiation to DE downregulates mitochondrial biogenesis regulators TFAM, POLG1 and POLG2, while upregulating PGC1-A, which results in increased mitochondria through upregulation of mtDNA transcription, replication, and mitochondrial membrane potential (ΔΨ). These alterations result in the maturation of the tricarboxylic acid (TCA) cycle gene transcripts. Elevated intracellular ATP levels provide energy for further differentiation and ROS generation as a result of OXPHOS. The activation of the electron transfer chain and TCA cycle promote the expression of enzymes involved in aerobic metabolism while down regulating glycolysis enzymes. Upregulation and activation of the oxidative glucose metabolism enables the activation of the triggering pathway of insulin secretion.

Differentiation of iPSCs into terminally differentiated β-like cells requires adaptations to meet new metabolic requirements. Several protocols have been reported to generate functional glucose-responsive insulin-secreting β-like cells. Following six-stages directed differentiation protocol, as previously described, new investigations also suggest addition of seventh stage for metabolic maturation with specific focus to reform mitochondrial metabolic dynamics in stage-specific manner (**Figure 3**) (97).

Differentiation of iPSC towards stage-1- definitive endoderm (DE) is the critical first step for differentiation towards pancreatic β cell lineage. It includes a variety of changes including molecular regulators- TFAM, POLG1/2, and PDGC1-A activation regulating mitochondrial fission and maturation. These factors affect mitochondrial mass, mtDNA abundance, mitochondrial ΔΨ, ATP production, intracellular ROS level, and expression of anaerobic and aerobic metabolic-associated genes (98). Differentiation to DE stage downregulates mitochondrial biogenesis, regulators mitochondrial transcription factor A and, mtDNA polymerase-γ -1 and -2, which results in increased mitochondrial mass through upregulation of mtDNA transcription and replication (99, 100). Furthermore, during DE differentiation, the expression of the mitochondrial biogenesis regulator peroxisome proliferator-activated receptor gamma coactivator 1- increased considerably, which is required for sustaining TCA gene expression (98). More recently, Jiang et. al., showed that mitochondria play crucial role in iPSC-derived definitive endoderm (DE) cell differentiation mainly by regulating ATP and ROS.

Mitochondria actively participate in DE differentiation to accomplish OXPHOS by producing ATP and ROS. If mitochondrial defects persist in human iPSCs then combined approach using ATP and nicotinamide can be useful to rescue optimal DE differentiation (101). Mitochondrial ΔΨ increases upon differentiation to DE and thereafter, suggesting the maturation of the respiratory chain which, in combination with increased mitochondrial mass, results in elevated intracellular ATP levels that provide energy for differentiation until stage-6 (98). Inhibition of TGF-β signaling disturbs the increase of ATP levels and hampers optimal differentiation.

At stage 4, generation of ATP is correlated with a sharp increase of mitochondrial ΔΨ and ROS levels because of upregulated OXPHOS during DE differentiation. The activation of the electron transfer chain and TCA promote the expression of enzymes involved in aerobic metabolism while downregulating glycolysis (102). HK1, HK2, phosphofructokinase and lactate dehydrogenase are remarkably downregulated during DE differentiation. Most glycolysis-related genes are downregulated upon DE differentiation except for GLUT3. On the other hand, OXPHOS-related genes were massively upregulated upon DE differentiation, including succinate dehydrogenase complex flavoprotein subunits A and B, the genes encoding succinate-ubiquinone oxidoreductase, and pyruvate dehydrogenase phosphatase catalytic subunit 1 and 2, encoding activating enzymes for the mitochondrial pyruvate dehydrogenase complex.

At stage 5, inhibition of TGF-β signaling using ALK5 inhibitor- RepSox interferes with the sharp increase in intracellular ATP and ROS levels, but also with the rearrangement of the metabolic transcriptome, thus impairing differentiation. Mature β cells rely on OXPHOS to meet energy demands and multiple genes encoding OXPHOS-related enzymes must be upregulated during the β cell differentiation process (103). Current differentiation protocols to generate stem cell-derived β (SC-β) cells yield a cell product able to secrete insulin in response to increasing concentrations of glucose and reverse diabetes upon transplantation in mice (104). However, *in vitro* GSIS response of SC-β cells is not comparable to that of cadaveric human donor islets in terms of the magnitude of insulin secretion or biphasic secretion profile. Metabolic assessment of human donor islets from patients with type-2 diabetes presented clear evidence for genomic regulation of OXPHOS-related genes controlling glucose responsive insulin release. It has been suggested that low expression of glycolytic genes (*LDHA*, *HK1*, and *PFKP*) and OXPHOS-related genes upregulation (*NDUFA5, NDUFA10, COX11*, and *ATP6V1H*) along with ATP production is essential for the activation of insulin secretion pathway (105). Although, glucose does not increase mitochondrial respiration in SC-β cells, studies reported that pyruvate stimulation facilitated increase in OXPHOS, which is indicative of immature metabolic characteristics (106). In depth comparative studies of mitochondrial machinery in SC-β cells have shown that decreased GSIS is not a result of a defective or immature cellular machinery or decreased insulin content, but metabolic failure because of imperfectly regulated differentiation (86).

**Figure 4:**
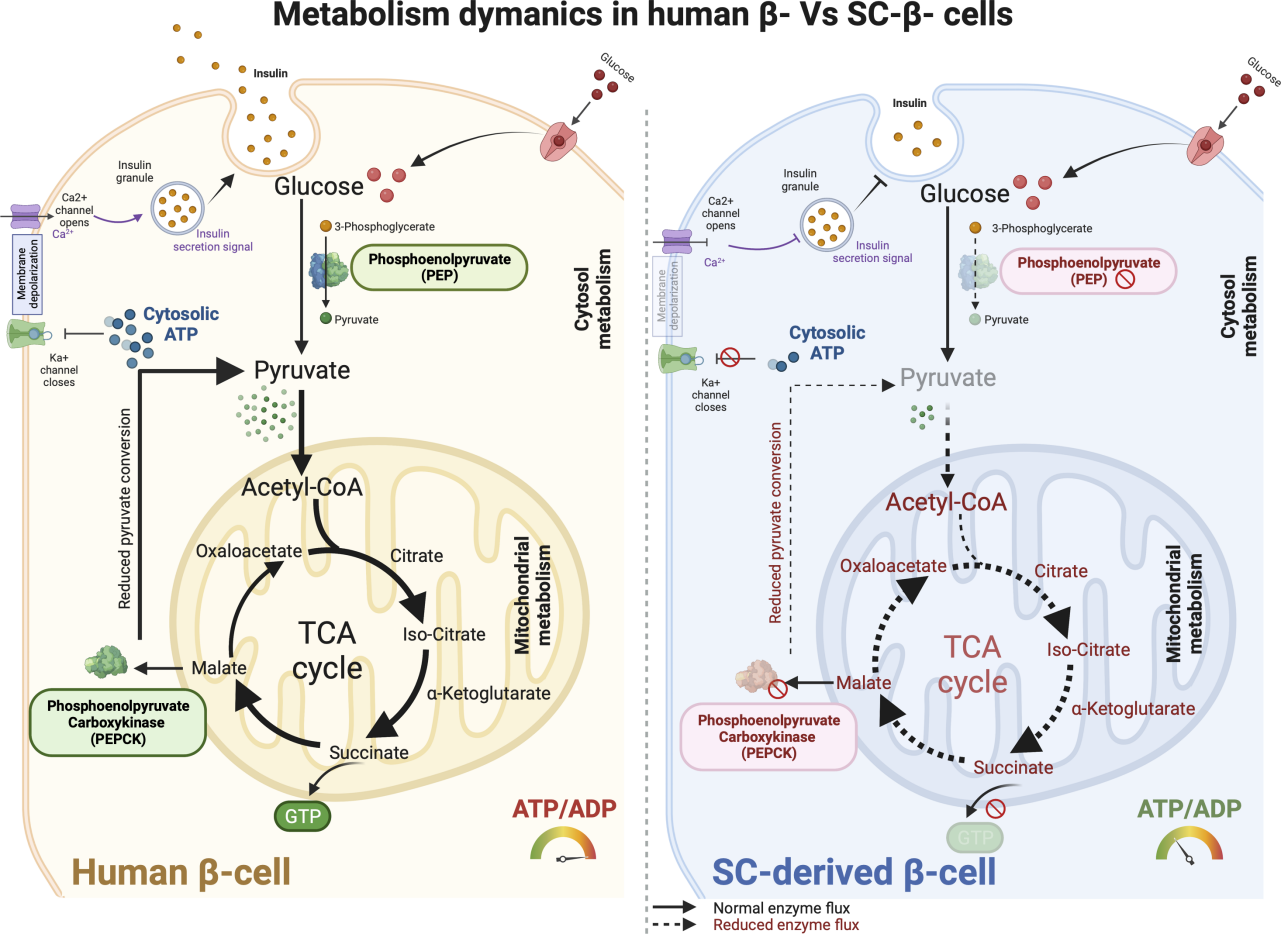
Metabolism dynamics in human primary β- cells and SC-derived β cells. The metabolic machinery in adult human β cells function optimally to sense glucose and express key metabolic enzymes that facilitate glucose-stimulated insulin secretion in response to insulin signaling and glucose transport. Glucose gets efficiently converted into pyruvate with phosphoenolpyruvate (PEP) activity which then enters mitochondrial membrane and serves as a precursor for TCA cycle respiration. Downstream enzymes converting GDP to GTP and phosphoenolpyruvate carboxykinase (PEPCK) allows continuous pyruvate flux using malate precursors which result in efficient ATP production. Increased cytosolic ATP pool then help facilitating Ka^+^ channel closure and Ca^2+^ depolarization to ensure glucose internalization and improved insulin secretion. Conversely, SC-derived β cells show anaplerotic cycling in the mitochondria. Substantial reduction in PEP activity reduces pyruvate production which further limit the mitochondrial respiratory cycle for enhanced ATP production. Further, lack of PEPCK activity results in dysfunctional mitochondria in SC-derived β cells. This overall shunt in metabolic precursors affect glucose responsive insulin production, metabolic control, and SC-β cell maturation like primary adult islets.

The enzymatic activity of SCS-GTP is decreased in SC-β cells as compared to β cells from donor islets, resulting in decreased GTP required for the activation of PEPCK-M, and hence, decreased generation of TCA cycle metabolites (**Figure 4**). Loss-of-function studies for SCS-GTP clearly mandate that mitochondrial preference to stimulate GSIS is SCS-GTP, despite functional SCS-ATP. GTP generated by SCS-GTP upon glucose stimulation is utilized by mitochondria isoform of PEPCK-M to synthesis PEP from oxaloacetate. This metabolic flux is revealed doubling of PEPCK-M flux to potentiate GSIS. PEPCK-M derived phosphoenolpyruvate comprises as much as 40% of the pyruvate that enters in the mitochondria to generate ATP; loss of PEPCK-M activity and its associated decreased in ATP production, and hence, decreased ATP/ADP ration, results in unsuccessful insulin secretory responses (107–109). Furthermore, SC-β cells present immature mitochondrial NADH shuttles, which also contribute to decreased GSIS (108). Upon transplantation and *in vivo* maturation, SC-β cells upregulate OXPHOS-related genes, which allows them to better respond to high concentrations of glucose *in vivo* (109).

Overall, differentiation towards definitive endoderm is the key step for successful generation of SC- β-cells and involves increasing the mitochondrial mass and maturation of the respiratory chain while downregulating glycolysis-associated genes. During the differentiation process, OXPHOS associated genes are required to be upregulated, which results in the generation of SC-β cells that rely on OXPHOS to fulfil their energy demands (103).

Several advanced and improved method for SC-islet generation have sought to understand how culture condition and chemical programming affect the metabolic phenotype *in-vitro* in direct comparison to primary β cells (86, 110–112). These studies presented a roadmap to highlight the key mechanisms responsible for SC-islet mitochondrial metabolic and functional (GSIS) immaturity. With protocol advancements, Hebrok and colleagues (110) in 2019 reported enriched clustering method to produce more functional SC-β cells that were reported to attain improved mitochondrial metabolic maturation with enriched OXPHOS, electron transport chain, TCA cycle metabolism and ATP biosynthesis. The study confirmed the presence of mature mitochondrial cristae that extended effective metabolic shift from glycolysis to OXPHOS in SC-β cells through the enrichment of ERRγ, a mitochondrial regulator of metabolic transition (110).

In parallel, Melton group performed a metabolic tracing study and reported that SC-islets primarily lack PEPCK-M activity due to slow glycolytic flux that prevented GTP sensing mechanolatry for glucose metabolism to PEP cycle. They also confirmed increased cytosolic ATP/ADP ratio. The study also reported to identify suppressed generation of PEP enzyme within the mitochondria due to glycolytic deficiency. Furthermore, differentiated SC-islets were observed to exhibit reduced GAPDH activity and the mechanism for this reduced activity remain elusive to conclude if this is due to post translational modification or other metabolic imbalance (*i.e* enzymatic conversion of glyceraldehyde-3-phosphate to 3-phosphoglycerate) (86). Further studies are required to ascertain these effects in scape-up generation of SC-islets.

More recently, Otonkoski group performed a detailed physiologic metabolic tracing study to characterize SC-islets metabolism against cadaveric donor islets using ^13^C labeled glucose (111) following optimal Balboa et al., protocol (106) for SC-islet generation. Mature β cells from donor islets exhibited augmented GSIS though glucose responsive metabolism into PPP, PSP and ABP pathways. SC-islets, however, showed remarkably lower enrichment of glucose-derived carbons entering into these pathways compared to primary islets. Key insights from this metabolic study suggested impaired mitochondrial TCA cycle metabolism in generated SC-islets, despite optimal directed differentiation. Furthermore, SC-islet metabolic function is dependent on core mitochondrial TCA cycle-driven metabolic with limited glucose-derived metabolic flux. The group inferenced that the primary reason for such impaired TCA metabolism might be due to more restrictive glycolytic metabolism. Collectively, these evidence indicate that although mitochondrial metabolic activity can be enhanced by limiting glycolysis, the TCA enzyme reactions and downstream metabolism (^13^C3 pyruvate and ^13^C5 glutamate) present in SC-islets are far beyond comparison to those seen in donor primary islets (111). Notably, deeper phenotypic investigations to correlate direct comparison of genomic to phenotypic datasets from donor islets and SC-islets, pre- and post-transplantation, are required to unravel metabolic bottlenecks in achieving optimal metabolic physiology.

## 7 Required metabolic traits for the generation of safe SC-β cells

To date, off target growth remains one of the major limitations for the translation of iPSC-derived therapies into the clinic. Mitochondria has been described to play an important role in the generation of teratomas and hence, several strategies have been studied to control the metabolic function of iPSCs and differentiated tissues to gain optimal graft function while controlling unwanted teratogenic growth. Herein, we summarize multiple approaches so far tested to refine ideal iPSCs and SC-β cell metabolism.

While the precise molecular mechanisms for the metabolic effect on iPSCs’ teratogenicity or differentiation remain undetermined, several studies have described the role of HIF1α in enhanced differentiation. Armstrong and colleagues showed direct evidence of the role of mitochondrial metabolism in iPSC to control teratoma formation rate when injected in SCID mice (20, 74). As a result of limited OXPHOS, iPSCs show reduced ROS scavenging genes, including glutathione reductase (*GSR*), *SOD2, MGSTI1*, and *MAPK26*, and hence, high levels of ROS result in senescence and apoptosis (43, 113). Thus, it might be beneficial to confirm the levels of ROS scavenging machinery in differentiated beta cells for OXPHOS dependency to circumvent the risk of teratoma post implantation. In general, iPSCs are reported to exhibit both low and high resting ΔΨ. Cells with lower ΔΨ show enhanced lineage specifications into germ layer cells. Schieke and colleagues injected several ESC lines into mice and showed that ESCs with lower resting ΔΨ show reduced risk of teratoma formation due to less oxygen consumption (114). This study confirms that the growth of rapidly proliferating cells is dependent on high aerobic glycolytic rates and nutrient availability. Therefore, differentiation of iPSCs with low ΔΨ into SC-β cells might allow to limit teratogenic growth.

Metabolically, SC-β cells exhibit distinct regulatory pathways to other lineages tissues such as SC-cardiomyocytes or neural progenitor cells (NPC). iPSCs produce most ATP through glycolysis while SC-cardiomyocytes produce most ATP using glucose, fatty acids, and lactate in OXPHOS (115) and NPCs rely predominantly on glycolysis rather than OXPHOS (116). Thus, differentiated cells can be enriched from iPSC favoring OXPHOS by using glucose and glutamine deprived media with lactate supplementation. This strategy, however, is not suitable for SC- β cells as glucose is required for insulin release; furthermore, β cells lack lactate transporter MCT13 and show reduced lactate dehydrogenase enzyme expression (117). Similarly, exposure to high concentration of certain amino acids (*i.e*., L-alanine and L-methionine) has been shown to selectively kill residual iPSCs (118, 119). This strategy cannot be used for SC-β cells either since prolonged exposure to L-alanine induces changes in the calcium responses promoting desensitization of insulin secretion, while L-methionine alters MafA expression without inducing teratogenicity.

In a recent study, Egli et al reported use of DNA replication inhibitor- Aphidicolin (APH) to be highly efficient in retarding teratogenic growth in SC-β implanted cells (120). The grafted cells produced c-peptide and reversed diabetes without any noticeable incidence of teratoma formation in long term engraftment. APH is a potent DNA polymerase inhibitor which has been shown to modulate mitochondrial DNA replication. The authors of this study clearly demonstrated that APH treatment of SC-islets completely eliminated cell growth, but they did not assess the impact of APH on mitochondrial replication or metabolism during the development of safe SC-cells (120).

Similarly, the mitochondrial redox protein Erv/ALR inhibitor- MitoBloCK-6, has shown potential in eliminating iPSCs in SC-β cells (121). Other identical and potential efficacious small molecules tested to influence metabolism in iPSCs are ER stress inducer- JC011, Substrate of ATP binding cassette transporter- 27-deoxy-27-oxookadaate, and pluripotency specific inhibitor PluriSln1 (122). JC011 and 27-deoxy-27-oxookadaate are reported to show neural cell toxicity while MitobloCK-6 shows cardiac cell toxicity. To date, only APH is evaluated for its toxicity in differentiated SC-β cells specifically, others including PluriSln1, JC011, 27-deoxy-27-oxookadaate, and MitoblockCK-6 remain untested for their application in differentiation protocols (121, 123, 124). Despite proven efficacy, almost none of these strategies has been tested for SC-β cells function, metabolism, and survival. There is a need to develop strategies to eliminate off target cells without compromising functionality and survival of SC-β cells to generate safe, off-target free SC-β cells and translate this approach into the clinic. We believe that further understanding of the metabolic function during iPSC reprogramming and differentiation will enable the optimization of differentiation protocols to produce GMP grade SC-cell products.

## 8 Concluding remarks

Mitochondrial metabolism plays a critical role in the generation of SC-islets, especially in the context of ongoing state-of-the-art differentiation protocols aimed at generating functional human pancreatic islet cells from pluripotent stem cells for cell therapy. These protocols are of great interest in the field of regenerative medicine for the treatment of diabetes, as they hold the potential to produce insulin-secreting cells that can replace damaged or dysfunctional pancreatic islets in diabetic patients. Here, we will provide an overview of mitochondrial metabolism in stem cell-derived islets and its relevance to the latest differentiation protocols.

Differentiation protocols for the conversion of human ES/iPSCs into functional SC-islets involve a series of carefully orchestrated steps that mimic pancreatic development *in-vitro*. These protocols typically include stages of definitive endoderm formation, pancreatic progenitor specification, and maturation into insulin-secreting β-like and other islet hormonal cells. The efficiency and success of these protocols rely on precise control of cellular processes, including mitochondrial metabolism. From the beginning of somatic cell reprogramming to SC-islet differentiation, cellular mitochondria undergo extensive remodelling for its morphological architecture, genomic elements and physiology balancing enzymatic machinery. Mitochondria, known as the "powerhouses" of the cell, are responsible for energy metabolism and generating adenosine triphosphate (ATP), the primary energy currency of the cell. In SC-islets, as in native pancreatic islets, mitochondrial metabolism plays a crucial role in energy production. During differentiation, stem cells transition from a relatively low-energy state to a high-energy state to support the demands of insulin secretion. This energy transition is effectively governed through a metabolic shift from glycolytic state to more energy producing OXPHOS metabolism.

Islet differentiation process confined to directed differentiation protocols involves metabolic shifts that are tightly regulated. In the early stages (definitive endoderm and foregut stages) of differentiation, cells rely more on glycolysis for energy production and gradually start transitioning into OXPHOs state as the mitochondrial machinery remodel. As the differentiating cells progress towards becoming more mature insulin-secreting cells, there is a rapid transition toward OXPHOS, which occurs in the mitochondrial TCA component. This shift is essential for meeting the increased energy demands associated with high insulin synthesis and secretion upon glucose stimulation. Furthermore, monitoring mitochondrial function is crucial in assessing the quality of SC-islets production with advanced protocols. Techniques such as measuring the oxygen consumption rate (OCR) can provide critical insights into the functional efficiency of OXPHOS competence and overall mitochondrial health. Optimal OCR is an indicative of mature functional mitochondria capable of meeting islet energy demands for regulated insulin secretion. Lastly, mitochondrial dynamics, including fission and fusion processes, are integral to maintaining a healthy mitochondrial population. Proper regulation of mitochondrial dynamics is essential for SC-islets to adapt to changing energy demands and maintain mitochondrial functionality.

While the protocol advancements have been made in understanding and optimizing cellular differentiation, mapping heterogeneity, transitional maturity and enhancing islet physiology for glucose control, robust mitochondrial metabolism in SC-islets remains a challenge. Achieving consistent and fully functional islet-like cells is a complex task to achieve and deeper phenotypic investigations are required to explore differentiation protocols, culture conditions, and genetic engineering approaches to enhance mitochondrial functionality and metabolism.

Reprogramming of cells into iPSC can provide a novel and unlimited starting cell source for personalized regenerative medicine. Reprogramming requires a transition in cellular characteristics, gene expression profiles and epigenetic status as well as major metabolic changes to sustain the highly proliferative status of iPSC. Understanding the metabolic demands and adaptations that iPSCs acquire from somatic cells are essential to tailor current protocols for inducing sustained reprogramming and identify the most optimal iPSCs that can deliver consistent cell products that can differentiate efficiently to provide safe cell therapy products. Overall, understanding of the metabolic demands of specific cell products require further investigation. Determining the molecular nature of metabolic immaturity and disconnect will advance the field by helping to resolve metabolic incapacities using small molecules and produce functional mature SC-β cells. Herein, we provide key recommendations for investigating and remodelling the mitochondrial metabolic adaptations for efficient and functional SC-islet manufacturing as closely comparable to native human donor islets. These approaches include **(a)** optimizing chemical differentiation factors to generate highly enriched endocrine progenitor population (PDX1^+^NKX6.1^+^CHGA^+^) that show reduced gene expression for critical disallowed genes like *LDHA, LDHB, HK1, HK2, IGF2, SLC16A1* (improve OXPHOS), and *ACOT7* (favor fatty acid metabolism and more ATP utilization), **(b)** incorporating extended maturation stage- Stage-7 by culturing SC-islets in growth factors-deprived and basal (5mM) glucose containing media to favor elevated ROS generation and ATP production and ultimately enhance OXPHOS transcriptome in SC-β cells, and **(c)** introducing factors (chemical or biological) for augmenting PEPCK expression and activity to channel glucose flux and co-factors required to shunt TCA metabolism and machinery, and **(d)** utilizing naïve transitioned iPSCs (favor bivalent metabolism) over primed iPSCs (favor glycolysis) for SC-β cell manufacturing. Naïve pluripotent stem cells have shown clear evidence for improved and defined multi-lineage differentiation (125–128) that attain functional maturity without the need for metabolic adaptation. Other strategies might include mitochondrial fusion regulation by Mfn1/2 expression to facilitate improved β cell function. In order to control Drp1, Mfn1/2 expression, and mTOR inhibition for potentiating insulin secretion and optimal metabolic functional result, it is crucial to create strategies that provide balance between mitochondrial fission and fusion in developing cells. Ultimately, screening human iPSCs for reported mtDNA mutation/ variants such as TRL-CAG1-7, Tfam and OPA1 gene defects may additionally provide critical insight for dysfunctional iPSCs.

The knowledge summarized in this review reflects key limitations and potential impediments in achieving essential mitochondrial features and optimal metabolic function in SC-β cells with an ultimate objective to produce metabolically active and safer product for clinical islet cell therapy.

## Author contributions

IJ and NC-G participated in study conceptualization, visualization, writing of the original draft, and final draft review and editing. KV participated in final draft review and editing. BM-G participated in final draft review and editing. ND participated in study conceptualization, visualization, writing of the original draft, final draft review and editing. Both corresponding authors supervised this project’s work, are responsible for the figures and text within the study, have ensured that all authorship is granted appropriately with all competing interests and disclosures identified, have ensured all authors have approved the work, have ensured adherence to all editorial and submission policies, and have ensured that figures accurately present the original data. AS participated in study conceptualization, funding acquisition, project administration, final draft review and editing and arbitrating decisions and disputes and ensuring communication with the journal. All authors contributed to the article and approved the submitted version.

## Glossary

**Table:**

2-OGDD	2-Oxoglutarate-dependent dioxygenases
αKG	Alpha-Ketoglutarate
ACC1	Acetyl-CoA carboxylase 1
AGEs	Advanced glycation end products
APH	Aphidicolin
ATG5	Autophagy-related protein 5
cMyc	Cellular Myc
DE	Definitive endoderm
DRP1	Dynamin-related protein 1
ETC	Electron transport respiratory chain
ESCs	Embryonic stem cells
ERR	Estrogen-related nuclear receptors
FAD	Flavin adenine dinucleotide
Fis1	Fission 1 protein
GLDC	Glycine decarboxylase
GLUT	Glucose transporter
GSIS	Glucose stimulated insulin secretion
GSR	Glutathione Reductase
HK	Hexokinase
HIF1	Hypoxia-inducible factor 1
iPSC	Induced pluripotent stem cells
JMJD	Jumonji C domain-containing family histone demethylases
Klf4	Kripple like factor 4
MAPK26	Mitogen-activated kinase 26
mtDNA	Mitochondrial DNA
MGST1	Microsomal glutathione S-transferase 1
NAD	Nicotinamide adenine dinucleotide
Nrf2	Nuclear erythroid-related factor 2
Oct4	Octamer binding transcription factor 4
OXPHOS	Oxidative phosphorylation
PARP1	Poly(ADP)Ribose Polymerase-1
PC	Palmytolcarnitine
PPP	Pentose phosphate pathway
PnPase	Polynucleotide phosphorylase
PDK1	3-Phosphoinositide-dependent kinase1
PDHK1	Pyruvate dehydrogenase kinase
PEPCK-M	Mitochondrial phosphoenolpyruvate carboxykinase
PK	Pyruvate kinase
PKM	Pyruvate kinase isoenzyme M
ROS	Reactive oxygen species
SC-β cells	Stem cell-derived β cells
SCS-GTP	GTP specific isoform of succinyl CoA-synthetase
Sox 2	SRY box transcription factor 2
Tcl1	T-cell leukemia/ lymphoma 1
TCA	Tricarboxylic acid cycle
TET	Ten-eleven translocation
